# Nebivolol Protects against Myocardial Infarction Injury via Stimulation of Beta 3-Adrenergic Receptors and Nitric Oxide Signaling

**DOI:** 10.1371/journal.pone.0098179

**Published:** 2014-05-21

**Authors:** Zheng Zhang, Liping Ding, Zhitao Jin, Guojie Gao, Huijun Li, Lijuan Zhang, Lina Zhang, Xin Lu, Lihua Hu, Bingwei Lu, Xiongjun Yu, Taohong Hu

**Affiliations:** Department of Cardiology, The Second Artillery General Hospital of Chinese People's Liberation Army, Beijing, China; Max-Delbrück Center for Molecular Medicine (MDC), Germany

## Abstract

Nebivolol, third-generation β-blocker, may activate β3-adrenergic receptor (AR), which has been emerged as a novel and potential therapeutic targets for cardiovascular diseases. However, it is not known whether nebivolol administration plays a cardioprotective effect against myocardial infarction (MI) injury. Therefore, the present study was designed to clarify the effects of nebivolol on MI injury and to elucidate the underlying mechanism. MI model was constructed by left anterior descending (LAD) artery ligation. Nebivolol, β3-AR antagonist (SR59230A), Nitro-L-arginine methylester (L-NAME) or vehicle was administered for 4 weeks after MI operation. Cardiac function was monitored by echocardiography. Moreover, the fibrosis and the apoptosis of myocardium were assessed by Masson's trichrome stain and TUNEL assay respectively 4 weeks after MI. Nebivolol administration reduced scar area by 68% compared with MI group (p<0.05). Meanwhile, nebivolol also decreased the myocardial apoptosis and improved the heart function after MI (p<0.05 vs. MI). These effects were associated with increased β3-AR expression. Moreover, nebivolol treatment significantly increased the phosphorylation of endothelial NOS (eNOS) and the expression of neuronal NOS (nNOS). Conversely, the cardiac protective effects of nebivolol were abolished by SR and L-NAME. These results indicate that nebivolol protects against MI injury. Furthermore, the cardioprotective effects of nebivolol may be mediated by β3-AR-eNOS/nNOS pathway.

## Introduction

Acute myocardial infarction (AMI), inducing permanent loss of cardiomyocyte mass and pathological left ventriclar remodeling, is one of the leading causes of death worldwide [1], [2]. Accordingly, finding novel and effective therapies is important to reduce myocardial injury induced by MI. In the heart, β-adrenoceptors (β-AR) are the primary regulator for cardiac performance, which are among the most widely used drugs for prevention and treatment of cardiovascular disease (eg, propranolol, which was the first β-blocker introduced into clinical practice) [3]. The cardioprotective effects of β1- and β2-AR are well established, including negative chronotropic and inotropic effects. In contrast to the well-characterized β1/β2-AR, accumulating evidence revealed that β3-AR also presents in the endothelium and myocardium [4]. Meanwhile, β3-AR stimulation regulates specific effects in the cardiovascular system [5], [6], [7]. Experimental studies have shown the stimulation of the β3-AR further activates endothelial nitric oxide synthase (eNOS) and increases NO release, which cause vasodilatation and improved endothelial function [8], [9]. Furthermore, it is well established that β3-AR is up-regulated in failing hearts, which was associated with attenuated left ventricular (LV) hypertrophy [10]. Therefore, β3-AR has been emerged as a potential target for the treatment of cardiovascular disease.

Nebivolol is the third-generation β-blocker approved by the Food and Drug Administration (FDA) for the treatment of hypertension. Given its greatest selectivity for cardiac β1-adrenergic receptors without intrinsic sympathomimetic activity, nebivolol reduces systemic vascular resistance and improving diastolic function [11]. Therefore, nebivolol was shown to reduce mortality and morbidity in elderly patients with heart failure [12]. In addition to its β1-blocking properties, previous studies have demonstrated that nebivolol also exhibits vasodilating properties by stimulating β3-AR [13]. Studies also confirm that β3-AR further activates nitric oxide synthase (eNOS) and increases NO release which exerts profound cardioprotective effects [14].

Although previous studied indicated that nebivolol could reduce cardiac remodeling and preserve cardiac function through β3-AR pathway, it is not known whether nebivolol administration plays a cardioprotective effect against MI injury. Therefore, we designed the present study to explore the potential role of nebivolol during MI and to elucidate the underline mechanism of their protective effects.

## Methods

### Animals

One hundred and fifty adult C57BL6/J mice (male, weighing 22 to 25 g) were housed in a temperature-controlled animal facility with a 12-h light/dark cycle. All procedures were approved by the Second Artillery General Hospital of Chinese People's Liberation Army Committee on Animal Care. (Approval ID: 2012-04) and were in compliance with Guidelines for the Care and Use of Laboratory Animals, as published by the National Academy Press. Mice were euthanized by cervical dislocation after anesthesia with 5% isoflurane.

Mice were randomly allocated into 5 groups with n = 30 each: (1) sham group (Sham); (2) MI group (MI); (3) MI + Nebivolol group (Nebivolol); (4) MI + Nebivolol+SR59230A group (Nebivolol+SR); (5) MI + Nebivolol+ L-NAME group (Nebivolol+L-NAME). After MI model, all of the above drugs or vehicles were administrated for 4 weeks respectively. Blood pressure was monitored with a tailcuff system (see Methods S1).

### Myocardial infarction model construction and Nebivolol administration

Myocardial infarction (MI) model was constructed by left anterior descending (LAD) artery ligation as previous described [15]. In brief, mice underwent aseptic lateral thoracotomy after anesthetized with 2% isoflurane. LAD was permanently ligated with a 6-0 suture. The ligation was deemed successful by characteristic ECG changes. Sham operated control mice underwent the same surgical procedures except that the suture placed under the left coronary artery was not tied.

Mice of MI+ Nebivolol group were administrated with Nebivolol at 2 µg/kg/hour via osmotic mini-pumps (Alzet Inc, Cupertino, CA) one day after MI operation. Mice treated with β3-AR antagonist were administrated with SR59230A at 0.1 mg/kg/hour via another osmotic mini-pump. Moreover, mice from Nebivolol+L-NAME group were treated with Nitro-L-arginine methylester (L-NAME) by intraperitoneal injection at 25 mg/kg for 4weeks after LAD ligation.

### Postmortem Histological determination of scar area

Mice were euthanized by cervical dislocation after anesthesia with 5% isoflurane for histological assay at 4 weeks after MI [15]. Hearts were embedded in paraffin after being fixed in 4% paraformaldehyde. Serial sections (5 µm thickness) were performed Masson's trichrome stain to detect scar area and fibrosis in cardiac muscle. Computerized morphometry was used to calculate the scar extent as the ratio of scar and total left ventricular area using Imaging Pro Plus software.

### Echocardiographic studies of cardiac function

Echocardiography was performed to assess the cardiac function after MI in a blinded manner [16]. At 2 days post operation (POD) and weekly until sacrificed, Mice were anesthetized (2% isoflurane and oxygen) and put in a supine position. Both two-dimensional and M-mode images were recorded using a 30-MHz transducer. Left ventricular systolic dimension (LVDs), left ventricular diastolic dimension (LVDd), anterior wall thickness (AWT) and posterior wall thickness (PWT) were measured to calculate left ventricular ejection fraction (LVEF) and fractional shortening (FS) as an average of three beats.

### *In vitro* apoptosis assay

Terminal deoxynucleotidyl transferase-mediated dUTP-biotin nick end labeling (TUNEL) assay was performed to evaluate myocardial apoptosis [15]. In brief, serial sections of heart tissue were stained with fluorescein-dUTP (In Situ Cell Death Detection Kit; Roche Diagnostics) for apoptotic cell according to the manufacturer's instructions. To identify all cell nuclei and myocardium, additional staining was performed using 4′,6-diamidino-2-phenylindole (DAPI) (Sigma) and the monoclonal antibody against Troponin I (cTnI, Santa Cruz). Randomly selected microscopic fields (n = 5) were evaluated to calculate the apoptotic index which was termed as the ratio of apoptosis (TUNEL positive) cells to total cells

### Western blot assay

4 weeks after cell transplantation, hearts were excised from peri-infarcted areas in LV for Western blotting assay following standard protocol [17]. Protein was isolated from homogenized LV tissue using cell lysis buffer (Cell Signaling Technology, Danvers, MA). Equal amounts of heated protein (100 µg) were separated by electrophoresis on 12% SDS-PAGE gels in a Tris/HCl buffer system, and sequentially electrophoretically transferred to nitrocellulose (NC) membranes. After blocking with 5% nonfat dry milk in Tris-buffered saline containing 0.05% Tween-20 (TBST), NC membranes were subjected to immunoblotting with appropriate primary antibodies at 4°C over night. Immunoreactivity was visualized by incubation with appropriate horseradish peroxidase–conjugated secondary antibodies and enhanced chemiluminescence kit (Amersham Bioscience, Buchinghamshire, UK). The densitometric analysis of Western blots was carried out using VisionWorks LS, version 6.7.1.

The following primary antibodies were used: β1-adrenergic receptor (1∶1000, Abcam); β2-adrenergic receptor (1∶1000, Abcam); β3-adrenergic receptor (1∶1000, Abcam); eNOS (1∶500,Cell Signaling Technology); Phospho-eNOS at Ser1177,Thr495, Ser114 (p-eNOS ^Ser1177^, p-eNOS ^Thr495^, p-eNOS ^Ser114^1∶500, Cell Signaling Technology); iNOS (1∶1000, Cell Signaling Technology); nNOS (1∶1000, Cell Signaling Technology) and β-actin (1∶ 5000, Abcam).

### Statistical analysis

All data are presented as mean ± SEM. Statistics were calculated using Prism 5.0 (GraphPad Software Inc, San Diego, CA, USA). Statistical comparisons for different groups were performed using one-way ANOVA followed by Student's paired, two-tailed t test for two groups' comparison. P values <0.05 were considered statistically significant.

## Results

### Nebivolol limits the extent of myocardial injury and decreases the mortality following MI

**Figure 1:**
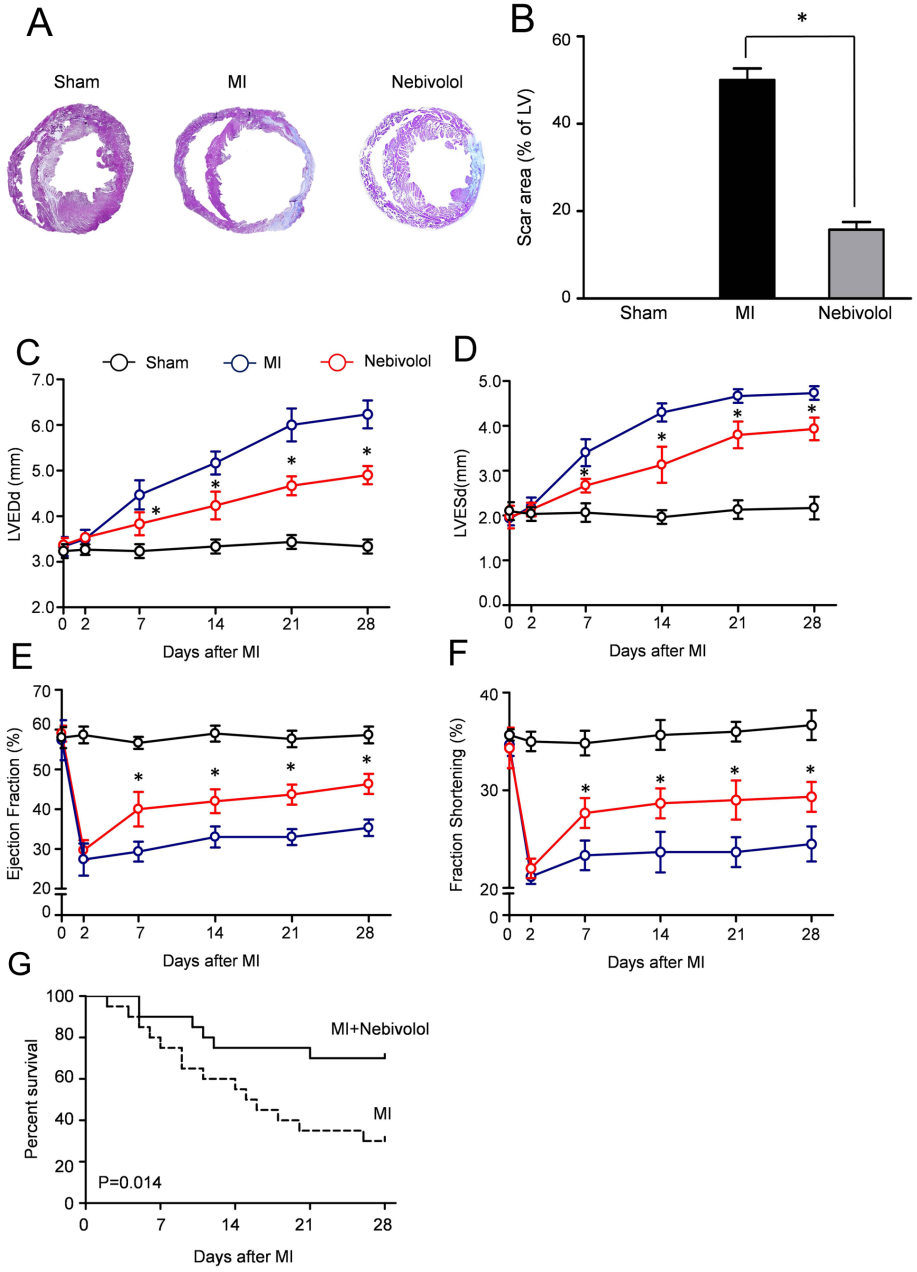
Effects of nebivolol on left ventricular fibrosis and heart function after MI. (a) Representative Masson's trichrome staining revealed left ventricular fibrosis 4 weeks after MI (magnification: 4x). (b) Quantitative analysis of the scar area (n = 10, *p<0.05). Quantification of left ventricular end diastolic diameter (LVEDd) (c), end systolic diameter (LVESd) (d), left ventricular ejection fraction (EF) (e) and fractional shortening (FS) (f) (n = 30, *p<0.05 *vs.* MI). Kaplan–Meier survival curves in MI group and MI+ nebivolol group (g).

To study the effects of nebivolol on the extent of fibrosis after MI, we performed Masson's trichrome staining. As illustrated in Figures 1a, severe fibrosis was observed in the hearts of MI group which was decreased by nebivolol administration. Quantitative measurement revealed that the scar area in MI group was 50.32±4.67%. Conversely, the scar area in mice receiving nebivolol was 15.75±4.75%, significantly less than that in MI group (15.75±1.75% vs. 50.32±2.67%, P<0.05).

**Figure 2:**
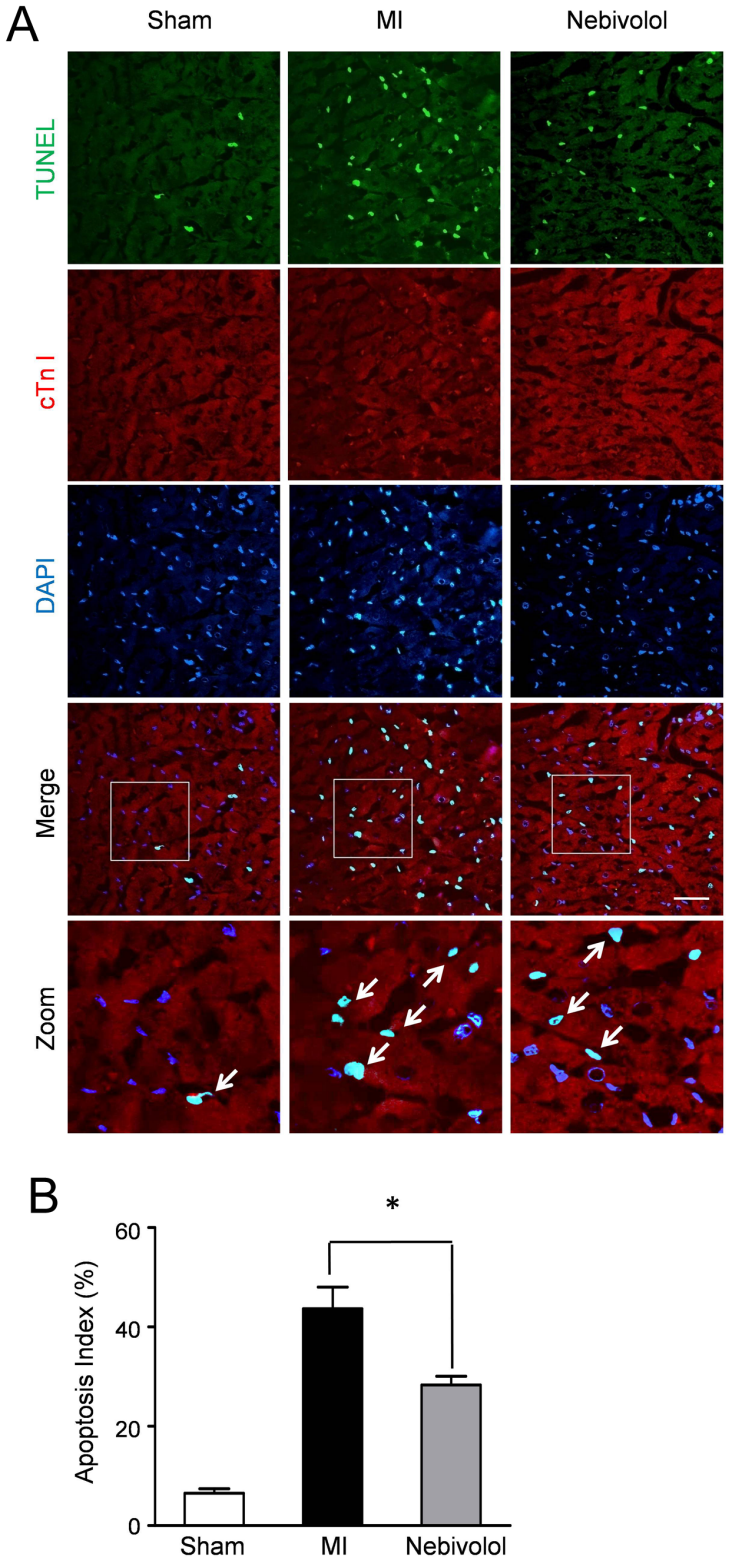
Nebivolol administration decreased cardiomyocyte apoptosis. (a) Representative photographs of TUNEL-stained heart sections from sham group, MI group and MI+ nebivolol group. Apoptotic nuclei were identified as TUNEL positive (green fluorescent). Myocardium was stained using a monoclonal antibody against Troponin I (red fluorescent) and total nuclei was stained by DAPI (blue fluorescent). Scale bar represents 50 µm. (b) Apoptotic cells were quantified by apoptotic index (AI) which was termed as the percentage of apoptotic cells. (n = 10, *p<0.05).

Furthermore, echocardiogram studies were performed to evaluate the cardiac function after MI in all groups. Serial echocardiographic analysis indicating that nebivolol administration manifested a trend towards improvement of cardiac performance after MI. Compared with sham group, MI increased the LVEDd and LVESd which were abolished by nebivolol treatment (Fig. 1c and 1d). Furthermore, the LVEF and FS were significantly enhanced in nebivolol group compared with MI group (Fig. 1e and 1f). These data suggested that nebivolol administration decreased fibrosis and preserved cardiac function after MI. Meanwhile, MI decreased the blood pressure, whereas increased the heart rate. However, nebivolol administration significantly increased the blood pressure, with decreased heart rate, compared MI group (Table S1). Moreover, the Kaplan–Meier survival curves (Fig.2g) indicated that nebivolol administration significantly decreased the mortality after MI.

### Nebivolol reduced cardiomyocyte apoptosis after MI

We next performed TUNEL staining to confirm the protective effect of nebivolol on the apoptosis of cardiomyocytes induced by MI. Representative immunofluorescence results in figure 2a revealed that apoptotic cardiomyocytes were more frequently observed in the MI group compared with Sham group or Nebivolol group. Quantitative analyses (Fig. 2b) demonstrated that the apoptosis index in MI group was 43.67±4.33%, significantly more than that in Sham group (6.54±0.89%, P<0.05). Conversely, nebivolol administration decreased the apoptosis of cardiomyocyte induced by MI (28.33±1. 76%, P<0.05 vs. MI).

### Nebivolol enhanced cardiac β3-AR expression after MI

**Figure 3:**
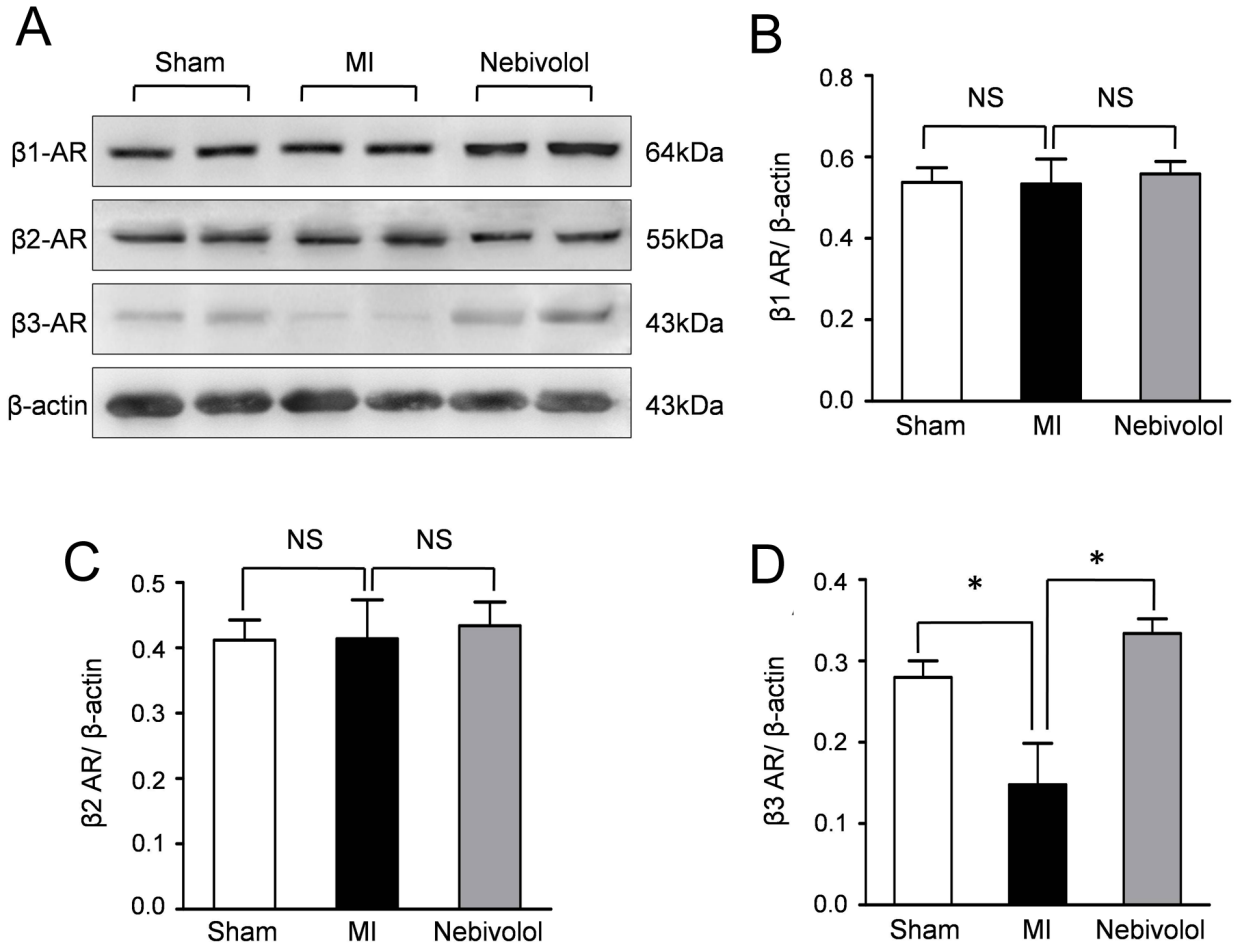
Effect of nebivolol on the expressions of β-AR subtypes. (a) Representative immunoblots of β1-AR, β2-AR and β3-AR in sham group, MI group and MI+ nebivolol group. The semiquantitative analysis of the expressions of β1-AR (b), β2-AR(c) and β3-AR (d) (n = 10, NS: non significance, *p<0.05).

Western blotting assay was performed to investigate the cardiac expressions of β-adrenoreceptor, including β1/β2-AR and β3-AR. Representative bloting results (Fig. 3a) indicated that the expression of β3-AR was decreased after MI which was abolished by nebivolol treatment. Semiquantitative analyses revealed that no changes in the expressions of β1-AR and β2-AR were observed in all groups (Fig. 3b,c). Furthermore, the expression ratio of β3-AR to β-actin in MI group was 14.80±2. 27%, significantly decreased compared with Sham group (28.03±2. 06%, P<0.05). Conversely, nebivolol treatment increased β3-AR expression (33.40±1. 78%, P<0.05 vs. MI) (Fig. 3d). These data indicated that the protective effect of nebivolol may be mediated by β3-AR.

### Nebivolol enhanced eNOS activation and increased nNOS protein expression

**Figure 4:**
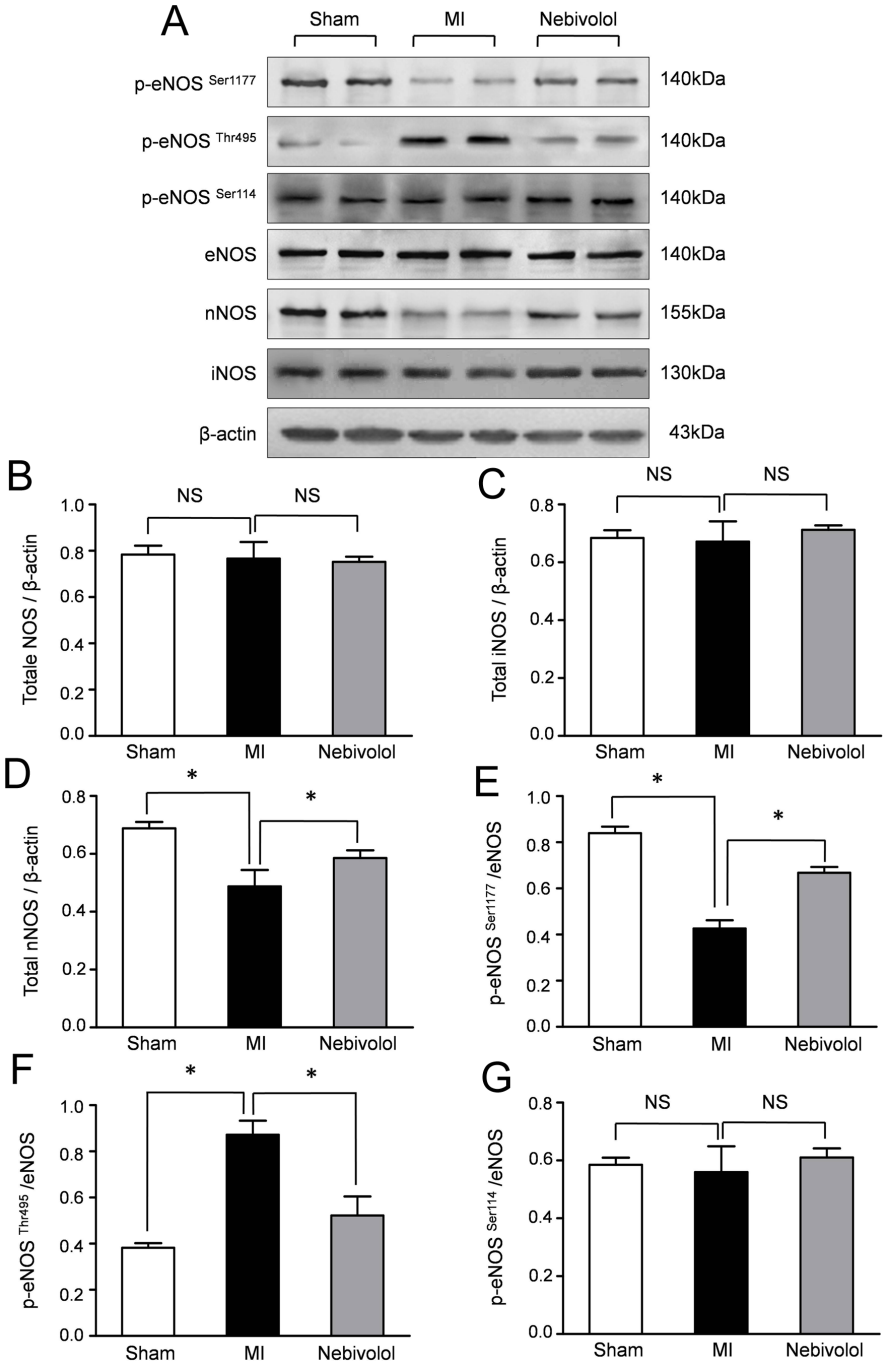
Nebivolol administration altered the phosphorylation status of eNOS and increased the expression of nNOS. (a) Representative immunoblots of *p*-eNOS (Ser1177/Thr495/Ser114), total eNOS, nNOS and iNOS in sham group, MI group and MI+ nebivolol group. Semiquantitative analysis of the expressions of eNOS (b), iNOS(c), nNOS(d), *p*-eNOS ^Ser1177^ (e), *p*-eNOS ^Thr495^ (f) and *p*-eNOS ^Ser114^ (g). (n = 10, NS: non significance, *p<0.05).

Previous studies suggested that β3-AR stimulation results in NO production via NOS which includes three isoforms (i.e. eNOS, nNOS, and iNOS). Therefore, we further performed Western blot assay to evaluate the effect of nebivolol on the expressions of NOS isoforms after MI. Representative bloting results and semiquantitative analyses (Fig. 4a–d) demonstrated that total eNOS and iNOS were unchanged in all groups. Conversely, the expression of nNOS was significantly decreased in MI group which was abolished by nebivolol treatment.

The activity of eNOS is generally modulated by three phosphorylation sites. Phosphorylation at Ser1177 activates eNOS, whereas phosphorylations at Ser114 and Thr 497 inhibit the eNOS activity.To furtherly investigate the role of nebivolol on the activation of eNOS, we examined the expressions of phosph-eNOS (p-eNOS). As representative results (Fig. 4a) and semiquantitative analyses in figure 4e–f, the expression of p-eNOS^Ser1177^significantly decreased, whereas the expression of p-eNOS^Thr495^ increased in MI group compared with Sham group. Furthermore, nebivolol administration resulted in an increase in p-eNOS^Ser1177^ expression with decreased eNOS phosphorylations at Thr 497 compared with MI. Interestingly, no difference of p-eNOS^Ser114^ expression was observed in all group (Fig. 4g).

### Cardiacprotective effects of nebivolol was abolished by β3-AR antagonism and NOS inhibition

**Figure 5:**
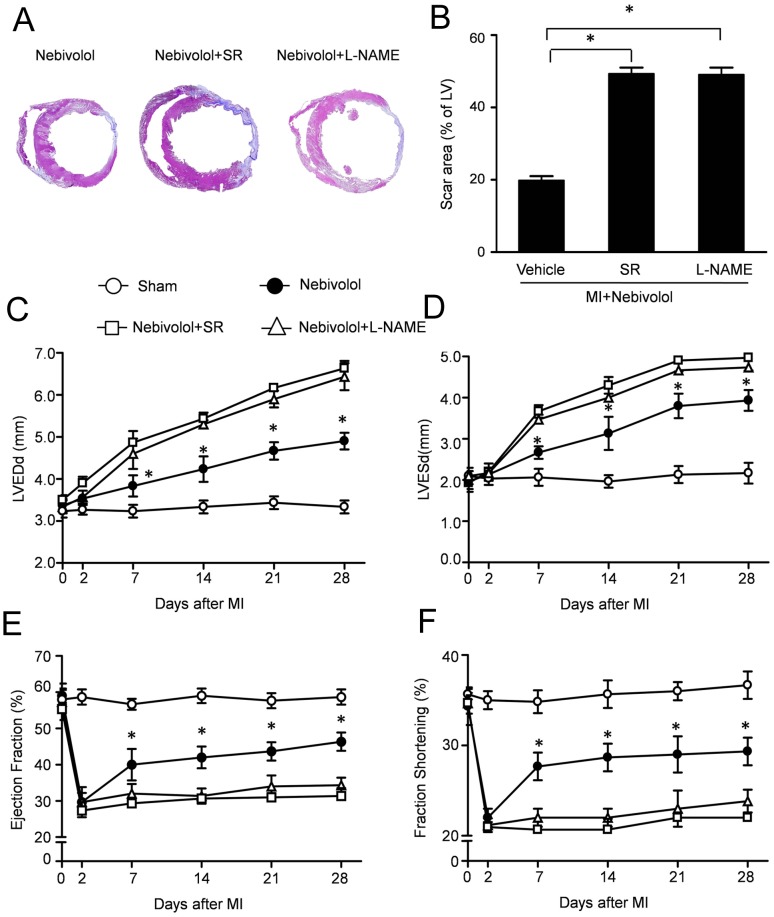
Cardiacprotective effects of nebivolol was abolished by SR59230A or L-NAME. (a) Representative Masson's trichrome staining 4 weeks after MI in Nebivolol group, Nebivolol+SR group and Nebivolol+L-NAME group (magnification: 4x). (b) Quantitative analysis of the scar area (n = 10, *p<0.05). Serial echocardiographic analysis was performed to assess the heart function in all groups, including left ventricular end diastolic diameter (LVEDd) (c), end systolic diameter (LVESd) (d), left ventricular ejection fraction (EF) (e) and fractional shortening (FS) (f). (n = 30, *p<0.05 *vs.* MI.)

SR59230A and L-NAME are well known as the inhibitors of β3-AR and NOS respectively. To illuminate the mechanism of the protective effect of nebivolol, either SR59230A or L-NAME was administrated with nebivolol. Representative Masson's trichrome staining and quantitative measurement in figures 5a,b revealed that the scar area in Nebivolol+SR group and Nebivolol+L-NAME group were 49.25±1.75% and 49.02±2.05% respectively, significantly higher than that in Nebivolol group (19.75±1.25%, P<0.05). Subsequently, echocardiogram studies were performed to evaluate the cardiac function (Fig. 5c–f). Serial echocardiographic analysis indicating that the LVEDd and LVESd in Nebivolol+SR group and Nebivolol+L-NAME group were increased, whereas the LVEF and FS were significantly decreased compared with that in Nebivolol group. These data suggested that the cardioprotective effects of nebivolol were abolished by specific inhibition of β3-AR and NOS.

### NOS inhibition and β3-AR antagonism abolished the anti-apoptosis effect of nebivolol

**Figure 6:**
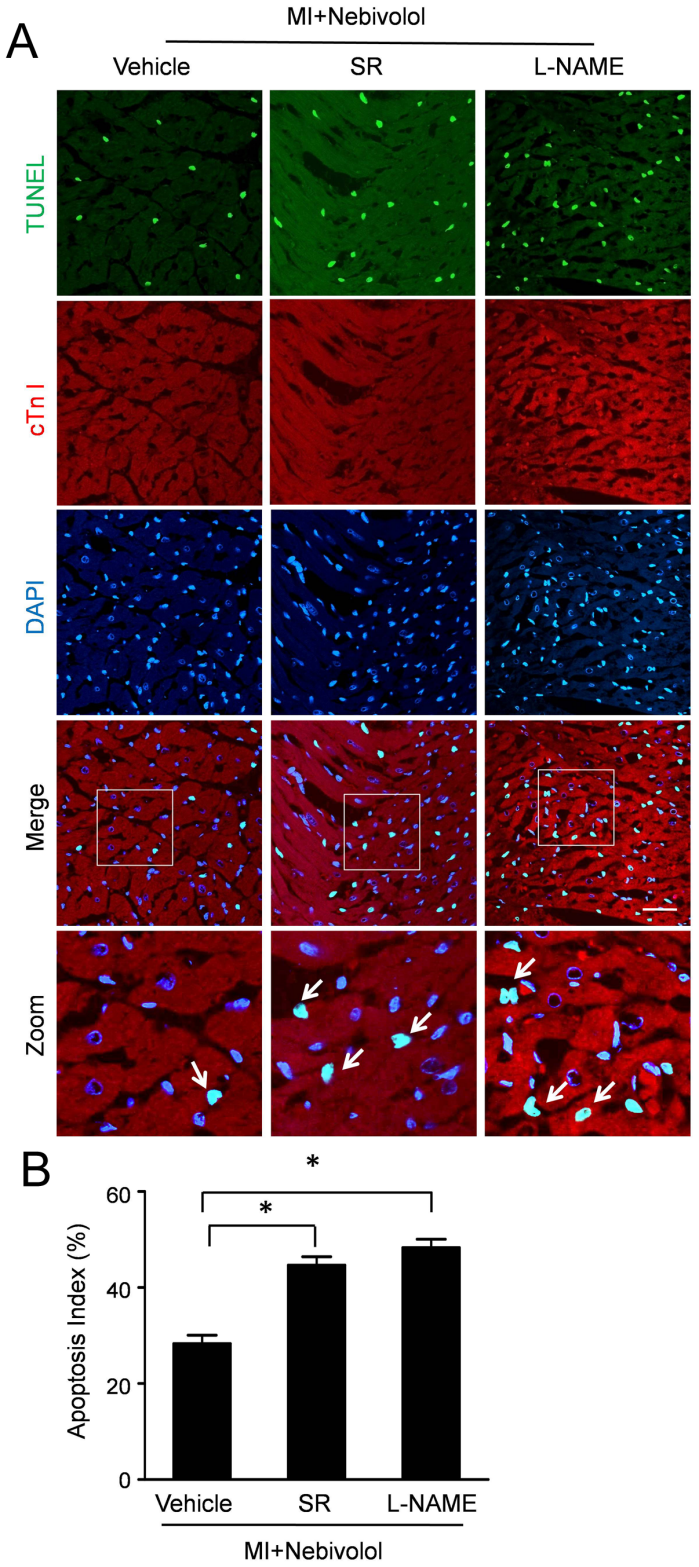
Anti-apoptosis effect of nebivolol was abolished by SR59230A or L-NAME. (a) Representative photographs of TUNEL-stained heart sections from Nebivolol group, Nebivolol+SR group and Nebivolol+L-NAME group (Scale bar: 50 µm). (b) Apoptotic cells were quantified by apoptotic index (AI) which was termed as the percentage of apoptotic cells. (n = 10, *p<0.05).

Representative TUNEL staining in figure 6a revealed that apoptotic cardiomyocytes were more frequently observed in Nebivolol+SR group and Nebivolol+L-NAME group compared with Nebivolol group. Quantitative analyses (Fig. 6b) demonstrated that the apoptosis index in Nebivolol+SR group and Nebivolol+L-NAME group were 44.67±1.76% and 48.33±1.85% respectively, significantly higher than that in Nebivolol group (28.33±1.65%, P<0.05).

### Nebivolol induced β3-AR signaling activation was abolished by SR59230A and L-NAME

**Figure 7:**
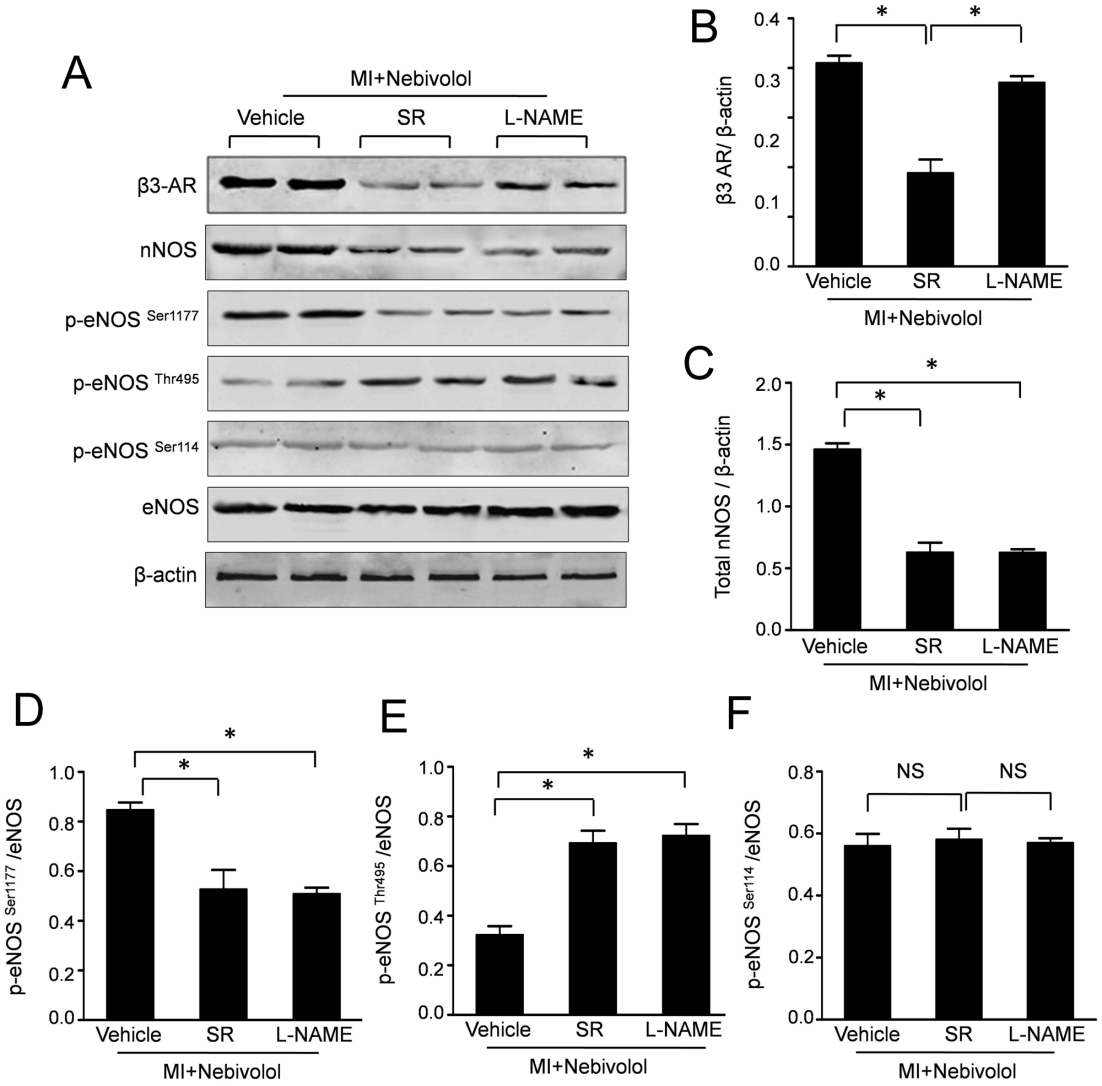
Nebivolol induced β3-AR signaling activation was abolished by SR59230A or L-NAME. (a) Representative immunoblots of β3-AR, nNOS, *p*-eNOS (Ser1177/Thr495/Ser114) and total eNOS in Nebivolol group, Nebivolol+SR group and Nebivolol+L-NAME group. Semiquantitative analysis of the expressions of β3-AR (b), nNOS(c), *p*-eNOS ^Ser1177^ (d), *p*-eNOS ^Thr495^ (e) and *p*-eNOS ^Ser114^ (f). (n = 10, NS: non significance, *p<0.05).

Representative bloting results and semiquantitative analyses (Fig. 7a–c) indicated that the expressions of β3-AR were decreased in MI+Nebivolol+SR group compared with that in MI+Nebivolol group. However, NOS inhibition with L-NAME had no effect on the expression of β3-AR. Furthermore, the expressions of nNOS in MI+Nebivolol+SR group and MI+Nebivolol+L-NAME group were significantly decreased compared with that in MI+Nebivolol group. The phosphorylations of eNOS were also detected by Western bolt assay. As representative results and semiquantitative analyses (Fig. 7d–f), the expressions of p-eNOS^Ser1177^ were significantly decreased in MI+Nebivolol+SR group and MI+Nebivolol+L-NAME group. Conversely, SR59230A or L-NAME administrated resulted in a significantly increase of p-eNOS^Thr495^ expression. Moreover, no difference of p-eNOS^Ser114^ expressions was observed in three groups.

## Discussion

In the present study, we found that nebivolol decreased the apoptosis of cardiomyocytes, and enhanced cardiac function after MI injury. Furthermore, the protective effects of nebivolol were abolished by β3-AR inhibition. Overall, this study demonstrates that nebivolol prevents cardiac dysfunction induced by MI via β3-AR-eNOS/nNOS pathway.

Nebivolol has recently been shown to cause peripheral vasodilatation, and provide beneficial effects on cardiac dysfunction. Sorrentino et al. [18] observed a significant increased EF% after treatment with nebivolol for 30 days after myocardial I/R injury. By contrary, Lefer et al. [19] failed to demonstrate significant improvements in cardiac function post-reperfusion after nebivolol treatment. In the current study, we confirm that nebivolol administration decreased the extent of fibrosis and reduced the apoptosis of cardiomyocyte, leading to an enhanced cardiac function recovery. Moreover, nebivolol administration decreased the mobility after MI. These results indicated that nebivolol exerts a cardioprotective role in the failing heart induced by MI.

The β-adrenoceptors, belonging to the G protein-coupled receptor super family, play an essential role in cardiovascular function. The β1- and β2-ARs, which mediates chronotropic and inotropic effects, are well established. Although the precise cardiac effects remain uncertain, β3-AR is of increasing interest due to its potential as a potential target for cardiovascular disease treatment [8]. Accumulating evidence suggests that β3-AR is activated at higher concentrations of catecholamines, and plays a negative inotropic effect antagonizing the effects of β1/2-ARs [20]. Moreover, previous study indicated that β3-AR plays a protective effect on exacerbated hypertrophy and cardiac systolic dysfunction induced by pressure-overload [21], [22]. All these literature support a significant role for β3-AR in the modulation of cardiac remodeling after MI.

Previous studies suggested that the protective effects of nebivolol are dependent on β3-AR stimulating [19]. However, the direct in vivo evidence has been lacking. In the present study, we observed that expression of β3-AR was decreased in the infarcted heart and nebivolol treatment increased β3-AR expression. Furthermore, the cardiacprotective effects of nebivolol were abolished by β3-AR antagonism SR59230A, indicated that β3-AR was involved in the protective effects of nebivolol.

Although the mechanism is not clarified, previous studies have demonstrated that the protective effects of β3-AR stimulation were inhibited by NOS inhibitor and could be reversed by excess NO production [9]. This evidence indicated that β3-AR plays a protective role by activating NOS pathway and NO release. However, three NOS isoforms (i.e. eNOS, nNOS, and iNOS) are involved in NO release which is involved in the regulation of myocardial function. There remains great controversy over which NOS isoform is the chief player in β3-AR signaling. Several studies have suggested that endothelial NOS (eNOS) is solely responsible for β3-AR induced negative inotropy. However, previous reports also revealed that the nNOS expression and nNOS-derived NO levels are both increased in failing hearts [8]. Moreover, it is an apparent paradox between the observations that eNOS-derived NO is decreased in failing myocardium, indicating the relative involvement of neuronal NOS (nNOS) in β3-AR modulates NO signaling.

Three phosphorylation sites, serine residue 1177 (Ser1177),threonine residue 497 (Thr495) and serine residue 114(Ser114), modulate eNOS activity. Phosphorylation at Ser1177 activates eNOS, whereas phosphorylations at Ser114 and Thr 497 inhibit the eNOS activity [23]. In the present study, we found that nebivolol treatment significantly increased p-eNOS^Ser1177^ and decreased p-eNOS^Thr495^ in the infarcted heart. Interestingly, the phosph-eNOS at Ser114 was unaltered with or without nebivolol administration, which was consistent with previous studies. The alterations of eNOS phosphorylation suggested that MI inhibited the eNOS activation which was abolished by nebivolol treatment. Emerging evidence demonstrated that nNOS-derived NO production plays a substantial role in regulating myocardial contraction [24]. Our results demonstrated that the expression of nNOS was decreased after MI which was abolished by nebivolol treatment. Consistently, previous studies revealed that administration of nebivolol or select β3-AR agonist CL 316243 both increased the expression of nNOS and reduced myocardial I/R injury.

Our study provides evidence that nebivolol treatment enhanced eNOS activation and increased nNOS protein expression, which was abolished by β3-AR antagonism SR59230A and NOS inhibitors L-NAME respectively. Moreover, nebivolol administration had no effect on the expression of iNOS. These results indicated that nebivolol acts as a dual activator of eNOS and nNOS to generate NO in the myocardium. Furthermore, the cardiacprotective effects of nebivolol were also abolished by L-NAME, indicated that nebivolol prevents cardiac dysfunction induced by MI by eNOS and nNOS which was activated by β3-AR pathway.

Although our study bears some clinical relevance, there are many limitations. First, the detailed physiologic and pathologic functions of β3-AR have not been extensively characterized. Moreover, there are still intense controversies about which NOS isoform is the chief player in β3-AR signaling. Further studies defining the exact mechanism are needed. In conclusion, we provided evidence that the administration of nebivolol has a substantial effect on recovery of heart function by eNOS and nNOS activated by β3-AR. These data collectively indicate that nebivolol administration has practical clinical use following myocardial infarction.

## Supporting Information

Methods S1 **Measurement of blood pressure.**
(DOC)

Table S1 **Hemodynamic parameters (heart rate and blood pressure) in different experimental groups.** Bpm: beats per minute. Data are mean±SD (n = 15). *P<0.05 vs. control group; †P<0.05 vs. baseline group.
(DOC)
