# Supplementary material for: Nebivolol Protects against Myocardial Infarction Injury via Stimulation of Beta 3-Adrenergic Receptors and Nitric Oxide Signaling
Source: PLoS One. 2014 May 21;9(5):e98179. doi: 10.1371/journal.pone.0098179 (PMC4029889; doi:10.1371/journal.pone.0098179)
Supplement: Methods S1 — Measurement of blood pressure. (DOC) [file pone.0098179.s001.doc]

**Methods S1**

**Measurement of blood pressure.**

Blood pressure was monitored with a tailcuff system (Noninvasive Blood Pressure System; PanLab). Briefly, rats were placed in a warm chamber (37°C) for 10 min to rest, and then occluding cuffs and pneumatic pulse transducers were placed on the tail. Five readings were obtained from each rat.
