# Supplementary material for: Nebivolol Protects against Myocardial Infarction Injury via Stimulation of Beta 3-Adrenergic Receptors and Nitric Oxide Signaling
Source: PLoS One. 2014 May 21;9(5):e98179. doi: 10.1371/journal.pone.0098179 (PMC4029889; doi:10.1371/journal.pone.0098179)
Supplement: Table S1 — Hemodynamic parameters (heart rate and blood pressure) in different experimental groups. Bpm: beats per minute. Data are mean±SD (n = 15). *P<0.05 vs. control group; †P<0.05 vs. baseline group. (DOC) [file pone.0098179.s002.doc]

**Table S1**

Hemodynamic parameters (heart rate and blood pressure) in different experimental groups

| **Characteristics** | **sham** | **MI** | **Nebivolol** | **Nebivolol+SR** | **Nebivolol+L-NAME** |
| --- | --- | --- | --- | --- | --- |
| **Baseline** | | | | | |
| Heart rate（bpm） | 623±25 | 611±17 | 629±12 | 613±21 | 631±28 |
| Blood pressure(mmHg) | 145±15 | 139±11 | 135±19 | 141±21 | 149±10 |
| **1 weeks after MI** | | | | | |
| Heart rate（bpm） | 618±17 | 641±11*† | 621±22 | 643±16*† | 639±18*† |
| Blood pressure(mmHg) | 139±21 | 121±13*† | 134±8 | 124±11*† | 119±13*† |
| **2 weeks after MI** | | | | | |
| Heart rate（bpm） | 628±11 | 649±13*† | 613±14 | 641±12*† | 632±11*† |
| Blood pressure(mmHg) | 141±13 | 117±22*† | 131±18 | 123±21*† | 121±14*† |
| **3 weeks after MI** | | | | | |
| Heart rate（bpm） | 622±14 | 642±15*† | 624±8 | 646±20*† | 639±15*† |
| Blood pressure(mmHg) | 133±23 | 122±14*† | 135±11 | 117±13*† | 119±19*† |
| **4 weeks after MI** | | | | | |
| Heart rate（bpm） | 627±7 | 644±9*† | 619±14 | 641±24*† | 647±11*† |
| Blood pressure(mmHg) | 143±16 | 116±12*† | 138±15 | 121±9*† | 129±14*† |

Data are mean±SD (n = 15). *P <0.05 vs. control group; †P< 0.05 vs. baseline group.
